# Mortality and heavy metals environmental exposure: a study in dogs

**DOI:** 10.3389/fvets.2023.1297311

**Published:** 2024-01-05

**Authors:** Roberta Giugliano, Maria Ines Crescio, Valeria Cosma, Valentina Ciccotelli, Barbara Vivaldi, Elisabetta Razzuoli

**Affiliations:** ^1^Istituto Zooprofilattico Sperimentale del Piemonte, Liguria e Valle d’Aosta, National Reference Center of Veterinary and Comparative Oncology (CEROVEC), Genova, Italy; ^2^A.Li.Sa., Piazza della Vittoria, Genova, Italy

**Keywords:** epidemiology, dog mortality, environmental exposure, cadmium, lead

## Abstract

**Introduction:**

Dogs are human companions and share environmental conditions with their owners. Epidemiological studies have shown that dogs seem to be good sentinel animals for the association of diseases and/or mortality provoked by chronic exposure to heavy metals (Cd, Pb).

**Methods:**

In the present work, we analyze the registered death cases and population from the National Canine Registry from 2020 to 2022, involving a dog population of 582,564 and 17,507 deaths. The mortality rate in male and not-purebred dogs is higher than in female and purebred dogs, respectively. The mortality cases were cross-referenced with the environmental pollution data relating to the concentration of Cd and Pb detected, between 2012 and 2022, in the various municipalities of the Liguria region. We then calculated SMR (Standardized Mortality Rate) throughout the region and found that mortality increases from the eastern to the western Ligurian coast.

**Results and discussion:**

We observed that the most polluted areas present the highest SMRs (IRR = 1.36, 95%CI: from 1.31 to 1.41). Considering dog ages, we found that mortality in young dogs is not affected by pollution, while mortality in old dogs (10–20 years old) is heavily affected by it (IRR = 8.97, 95%CI from 8.09 to 9.93). In conclusion, the data suggest the importance of canine health and biomonitor studies and provide a basis for future research involving both animal and human health.

## 1 Introduction

Dogs have been domesticated for thousands of years and have become an integral part of human society, serving as companions, workers, and guardians and sharing environmental conditions with human beings. Some authors (1) have reported that the remaining life period of dog owners could realistically be calculated through knowledge of their dog’s life expectancy. To date, most canine death literature are based only on pets’ owners statements collected during surveys (2, 3) and dog adoption databases obtained from referral or first-opinion veterinary caseloads (4, 5) or insurance databases (6). Due to collection difficulties, which often occur in animal registries, epidemiological studies involving animals are rare (7).^1^

Domestic animals share the same outdoor environment as humans and are therefore exposed to outdoor pollutants. Unlike humans, pets do not engage in occupational activities or lifestyle habits such as smoking or consuming alcohol, which could confound the interpretation of epidemiological findings. A case–control study was conducted in the “triangle of death” region near Naples, based on data obtained from the Cancer Registry of the Sanitary Local Unit Naples 4 (8), to investigate the link between pollution and mortality/tumors. The study examined the incidence of tumors in two macro areas, one classified as “low risk” and the other as “high risk.” The authors found that animals living in the most polluted area had a 55% higher risk of developing tumors, particularly lymphoma, with a 72% higher risk. Another recent study conducted in Italy identified neoplastic disease as the fourth leading cause of death in dogs (9), raising the possibility that environmental exposure may play a role in canine mortality.

Environmental monitoring has traditionally been conducted using non-living matter, but recent advancements have led to the use of living organism samples, known as “biomonitors.” Among the most widely used living proxies, moss serves as a key biomonitoring matrix for the temporal assessment of trace element air pollution levels. Moss biomonitoring has been applied to air pollution studies in the Republic of Moldova as part of the International Cooperative Program on Effects of Air Pollution on Natural Vegetation and Crops (UNECE ICP Vegetation) (10–13). According to a report by the Ministry of Environment and Natural Resources (11, 14), the concentration of heavy metals in precipitation is correlated with their concentration detected in soil. Therefore, moss biomonitoring is considered the primary matrix for assessing the pollution level of trace elements in the air over time. Another common living proxy for environmental monitoring is sylvatic animals that consume mainly undergrowth matter and can bioaccumulate heavy metals in their target organs. In long-term studies, living organisms could provide a better understanding of environmental impacts over time by comparing environmental matrices. Analyses on living organisms can summarize all biological, chemical, physical factors monitored using environmental matrices and can give us an holistic overview of environmental changes (10–13). Thus, *biomonitors* provide a more comprehensive assessment of the ecosystem compared to monitoring based solely on environmental matrices. This peculiarity is pivotal in gradual and cumulative changes in the environment. A study conducted in six hunting areas in Viterbo (Italian Province) demonstrated that wild boars (WB) are more exposed to heavy metal contamination than farm animals due to their diet and habits, and they therefore represent a good environmental proxy. In WB, median values of heavy metals were slightly greater (0.125 mg kg^−1^) than in farm animal livers (0.112 mg kg^−1^) (15). This trend was confirmed by other risk-assessment studies that showed that health risks related to heavy metal exposure are greater for hunter populations than for the general populace (16). In addition, the reduction of habitats, climate change, and dietary habits has led to an increased presence of WB in recent years (17–20), and recreational hunting of WB and the consumption of their meat further provides direct human contact with WB (20–22). Therefore, we selected WB as an indicator of human exposure to pollutants, as already reported in the literature (20).

In this work, metal pollution information was obtained by wildlife biomonitors sampled within the Ligurian territories. Liguria is one of the smallest Italian regions (5,418 km^2^) and is bordered to the north by the Apennine Mountains and to the south by the Ligurian Sea. This unique geographical conformation entails the close proximity of rural areas and cities, along with a miscellany of pets and sylvatic animals, which, in turn, share the same environmental stressors. Thus, consistent with scientific literature (10–13), metals extracted from WB organs (liver and kidney) provide an overview of the metal exposure of pets and humans.

Environmental exposure to cadmium could be due to food and water heavy metal contamination (23). Global anthropogenic atmospheric cadmium emissions are primarily released from non-ferrous metal smelting, coal burning, and non-metallic mineral manufacturing and have increased from 1,679 tons to 2,246 tons during the last century (24). The increase in emissions has resulted in higher concentrations of cadmium in topsoil and vegetation, leading to increased human exposure (25). The health risks associated with cadmium are the outcome of a series of prolonged and continuous processes that link heavy metal emission sources to human exposure and accumulation, as described in previous studies as the “Cd transfer continuum” (24).

Cadmium is now recognized as a global public health hazard due to its persistent presence in the environment and its extended biological half-life (26). High intake of cadmium through the diet, similar to other heavy metals, can cause functional disturbances. Such effects are particularly severe in children, who absorb metals more efficiently than adults and are biologically and developmentally more sensitive to these effects (27).

As reported by other authors, Cd^2+^ leads to an immunosuppression status and negatively affects the immune function of monocyte-derived macrophage (moMФ) cells, increasing susceptibility to infection (28).

Cadmium induces the synthesis of metallothionein, a cysteine-rich protein that can transport cadmium to target organs such as the kidneys and liver and binds to metabolites in tissues (29) causing cell damage (30). Cadmium also accumulates in the liver in its free form, where it reduces glutathione synthesis and promotes oxidative cell damage, leading to apoptosis and extensive hepatocellular necrosis (31, 32). The accumulation of cadmium can also interfere with vitamin D and calcium metabolism, resulting in bone demineralization, which ultimately leads to bone loss (33, 34). Furthermore, cadmium has been implicated in the pathogenesis of canine and human epilepsy or seizures (35) and chronic kidney disease (24, 36) and in the development of breast cancer (37).

Lead exposure has two main sources: food intake and coal combustion, with the latter being a significant source of lead exposure for children and domestic dogs (38). A recent study conducted in Australia demonstrated that during seasonal deer hunting, dogs are more exposed to heavy metals due to their consumption of game meat instead of common industrial feed (39). Hounds are typically fed low-quality meat cuts, also known as “trimmings,” which are closer to the bullet wound tract, compared to the cuts consumed by human hunters (primarily loin/backstraps and cuts from the hind legs) (40, 41). Once absorbed, it interferes with various biochemical reactions and cellular structures, causing gastrointestinal and neurological damage of varying degrees depending on the exposure duration and the species affected. Intoxication manifests as abdominal pain and diarrhea and can also lead to neurological damage, such as depression, ataxia, convulsions, and even death (42, 43). Furthermore, lead accumulation in the kidneys can cause proximal tubular nephropathy (42). Lead contamination in food has serious consequences for human health, and attention has recently been focused on lead toxicity in children (44, 45). Evidence suggests that cognitive development is impaired in children exposed to lead (46, 47). Lead exposure in dogs can lead to other health problems, including neurodevelopmental impairment and anemia (48) and a recent study has reported that dogs with high hepatic lead concentrations exhibit microcytosis (49).

Previous research has established the need for the ongoing monitoring of heavy metal levels in food and the environment, with a particular focus on vulnerable populations, such as animals and humans (50). However, to the best of our knowledge, there has been limited exploration of the relationship between mortality in dogs and exposure to environmental pollution in existing literature. While some recent studies have examined heavy metal tolerance in animals (51) or identified common causes of death in dogs (9), none of these relate to the association between pollution data and the development of canine diseases.

Owing to the above, the aim of this study is to investigate whether there is an association between the mortality of dogs and environmental pollution by observing biomonitors of heavy metals in animals, such as WB, which are prevalent throughout the region and using the data from the National Canine Registry.

## 2 Materials and methods

### 2.1 Data collection and handling

#### 2.1.1 Data were collected and handled by the partner institutions

Animal incidence data were derived from death cases and the population-based National Canine Registry. Individual information on dog breed, sex, neutered/spayed status, date of birth and death, and regional territorial unit code of the town of the owner’s residence were collected from 01 January 2020 to 31 December 2022. In this study, only Ligurian municipalities were studied. Some death information was censured and for this reason, individuals older than 20 years were reported as deaths by default. This censoring method is very common in animal registers (8, 9).

Environmental data were obtained from the Istituto Zooprofilattico Sperimentale del Piemonte, Liguria e Valle d’Aosta (IZS PLVA) database. Environmental data consisted of biomonitor data obtained by extracting the concentration of heavy metals (Cd and Pb) from target organs of WB between 2012 and 2022, which was conducted by the Chemical Unit of the Istituto Zooprofilattico Sperimentale del Piemonte, Liguria e Valle d’Aosta. All WB were passively and actively sampled and came from the Ligurian territory.

### 2.2 Chemical analysis of metals from WB organs

Tissue samples were homogenized and then transferred to a Teflon^®^ microwave vessel and mixed with 65% nitric acid (Sigma-Aldrich S.r.l., Milano, cat. V001338) and hydrogen peroxide (Merck Millipore, Germany, cat. 1.086.001.000). The samples were then digested using a laboratory microwave oven. The extract was filtered and diluted with ultrapure water. Determination of Cd^2+^ contents was carried out using the Analytical Yena 650 Plus Atomic Absorption Spectrometer with graphite furnace, at 228.8 nm, with a current of 4 mA. Quantification was obtained by the standard addition method, adding a certified standard solution purchased from Ultra Scientific to the matrix solution. The data were plotted as absorbance versus the amount of the standard added. The least squares line intersects the x-axis at the negative of the concentration of the sample. The quantification limit (LOQ) was equal to 0.020 mg/kg. To test reagent purity and possible contamination, “blanks” were analyzed for each run using the procedure described as follows.

### 2.3 Data analysis

For the current study, age was obtained by subtracting the date of birth from the date of death and categorized into six age groups (4–5 years, 6–7 years, 8–9 years, 10–11 years, 12–13 years, 14 years and more), representing the entire population. For the sole purpose of performing the risk analysis, obtaining a more robust model, mortality rates were stratified by three age groups (4–5 years, 6–9 years, and 10–20 years). Breeds were further classified in a dichotomous variable (purebred, not purebred).

A descriptive analysis of the data was carried out, taking into consideration calculated death age groups, sex, and owners’ city of residence for all 3 years of the study. Younger dogs (0–3 years old) were ignored as their exposure time was relatively short and there are various other factors responsible for animal death, such as hypothermia and birth weight (52, 53). The neutered/spayed status was not considered as no information on sterilization date was available.

The indirect standardized mortality ratio (SMR) was estimated as the ratio of observed deaths to expected deaths. Expected cases were estimated using regional rates stratified by sex (male/female), dichotomous breed variable (purebred, not purebred), and municipalities.

Environmental pollution was monitored considering cadmium and lead concentration separately. Descriptive maps were created classifying cadmium and lead concentrations according to the cadmium meat EU limit (Regulation (EC) No 853/2004 (54), Commission Regulation (EC) No 1881/2006 (55)).

To further understand the factors that contribute to dog mortality, we developed a Poisson GLM (Generalized Linear Model) considering pollution level, sex, breed, death age group, and observation year as covariates. Models were estimated using the stepwise approach. Poisson is a statistical technique commonly used to model count data, such as mortality rates, and can account for the effects of multiple predictor variables. In our model, we included cadmium and lead concentrations (below or above the EU limits) and we adjusted for confounding variables, namely, sex, breed, and age. All analyses were carried out using STATA 17.0 (StataCorp, Texas, United States).

## 3 Results

### 3.1 Biomonitor dataset

Considering all samples processed from 2012 to 2022, the cadmium concentration average obtained was 0.53 ± 0.40 mg/kg and the lead average was 0.98 ± 0.21 mg/kg. Samples with a cadmium concentration higher than 0.50 mg/kg and lead higher than 0.10 mg/kg were considered contaminated (as reported in EC Reg. 1881/2006 for the liver of cattle, sheep, pigs, poultry, and horses) (55).

In the biomonitor dataset, more than 90% of the total Ligurian region was covered, meaning that our biomonitor dataset is significant in monitoring environmental pollution (Table S2, Supplementary material). Among all the municipalities where heavy metals were investigated, cadmium concentrations were above the EU limit for 72 municipalities and below the limit for 113. Regarding lead concentration, all municipalities presented concentrations above the EU limit. No heavy metal information was available for 50 municipalities.

**Figure 1:**
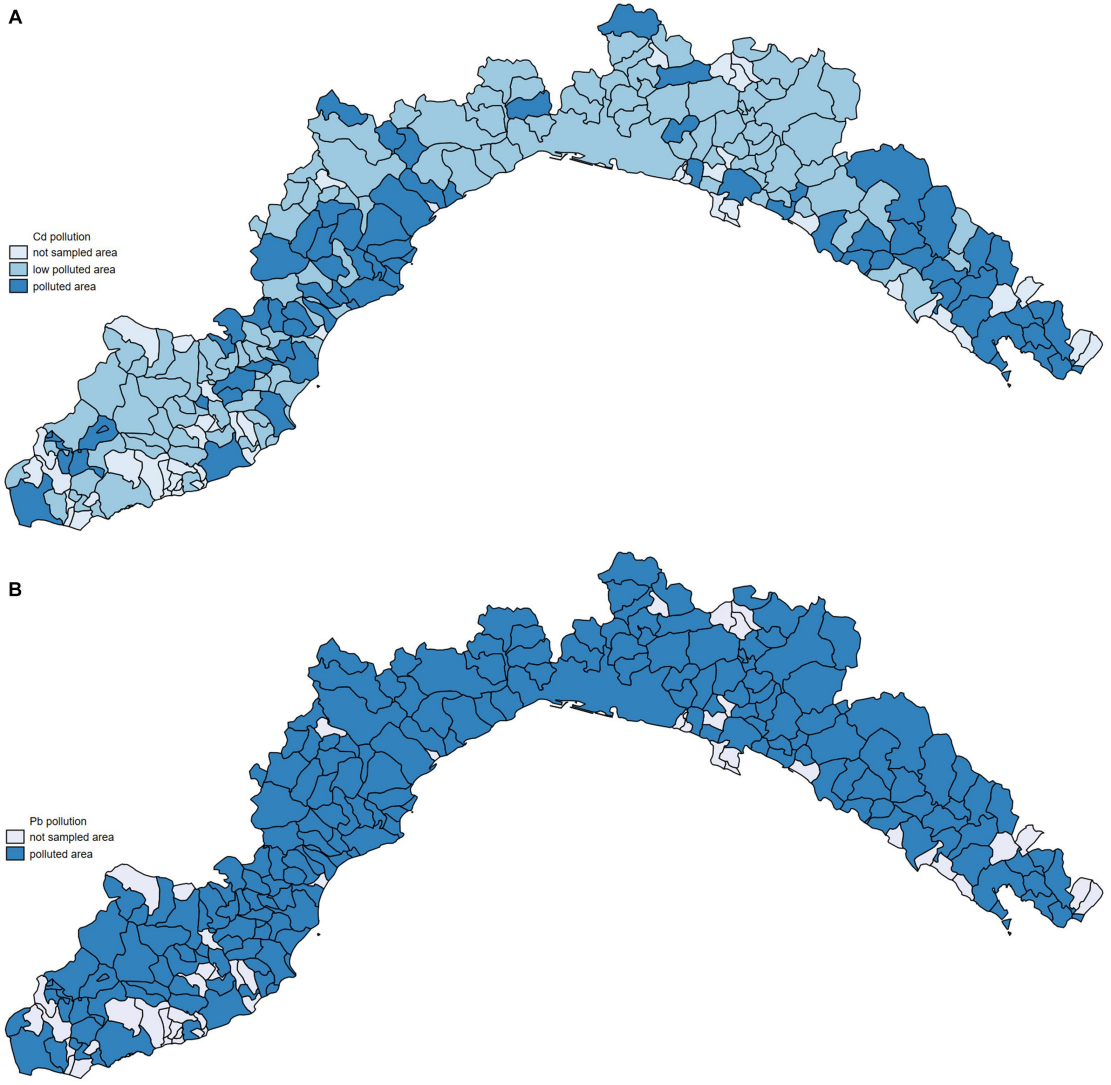
Heat map of Ligurian cities representing cadmium **(A)** and lead **(B)** concentrations. Legend: not sampled area (municipalities where no heavy metal data were available), low polluted area (cadmium <=0.50 mg/kg; lead <=0.10 mg/kg); polluted area (cadmium >0.50, lead >0.10).

The level of pollution at the provincial level was then evaluated and the median cadmium and lead concentration for each Ligurian province was calculated considering the entire period between 2012 and 2022. Cadmium mean in the Genova province was 0.38 mg/kg (95%CI: from 0.27 to 0.49), in Imperia, it was 0.38 mg/kg (95%CI: from 0.27 to 0.49), in La Spezia, it was 0.79 mg/kg (95%CI: from 0.60 to 0.98), and in Savona, it was 0.64 mg/kg (95%CI: from 0.38 to 0.90). Regarding lead, the mean obtained in Genova was 0.33 mg/kg (95%CI: from 0.39 to 0.27), in Imperia, it was 0.35 mg/kg (95%CI: from 0.42 to 0.28), in La Spezia, it was 0.71 mg/kg (95%CI: from 0.86 to 0.56), and in Savona, it was 0.12 mg/kg (95%CI: from 0.65 to 0.41). What emerges from the data is that municipalities within the La Spezia and Savona provinces represent the Ligurian areas with the highest cadmium concentrations (Figures 1A,B).

### 3.2 Individual data

**Figure 2:**
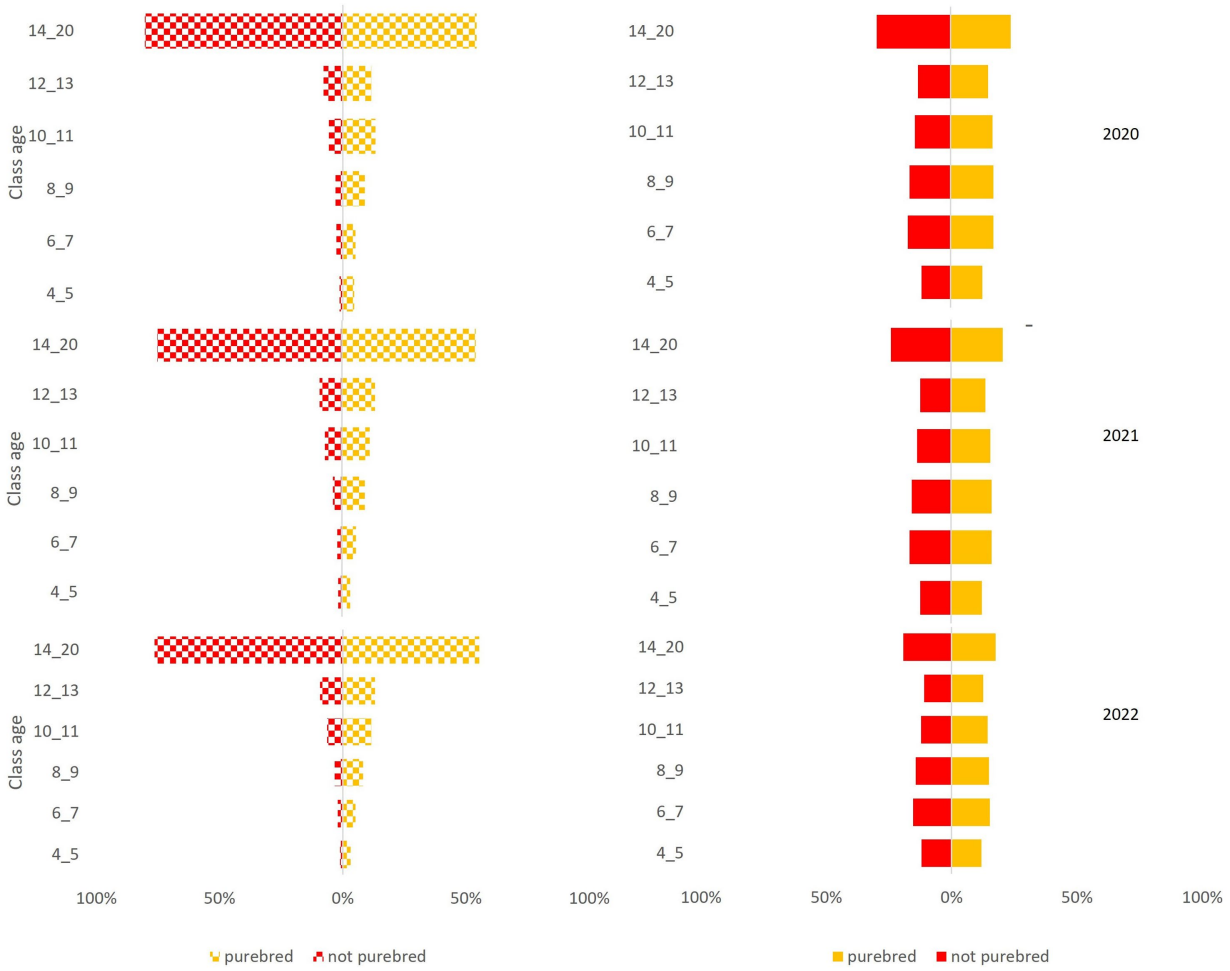
Pyramid graph of the deaths (on the left) and the canine population (on the right) over the 3 years (2020–2021-2022) stratified by breed (purebred, not purebred) and age group (4–5 years, 6–7 years, 8–9 years, 10–11 years, 12–13 years, 14–20 years).

**Figure 3:**
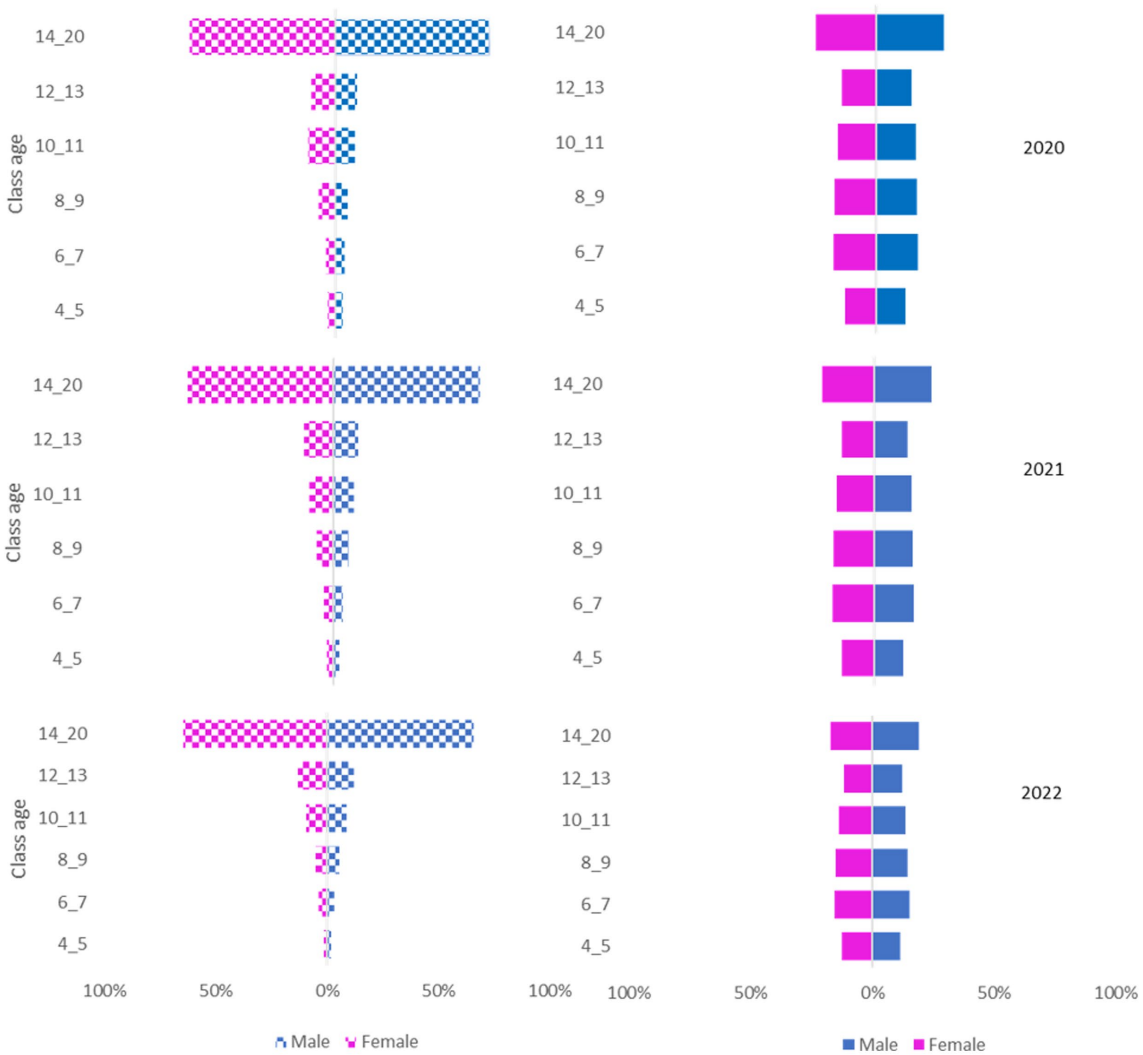
Pyramid graph of the deaths (on the left) and the canine population (on the right) over the 3 years (2020–2021-2022) stratified by sex (female/male) and age group (4–5 years, 6–7 years, 8–9 years, 10–11 years, 12–13 years, 14–20 years).

The National Canine Registry database recorded 17,507 deaths from a total population of 582,564 in the Ligurian area from 2020 to 2022. Gender distribution was equal (49% female dogs; 51% male dogs) and remained constant throughout the three years of the study period (Figures 2, 3).

**Figure 4:**
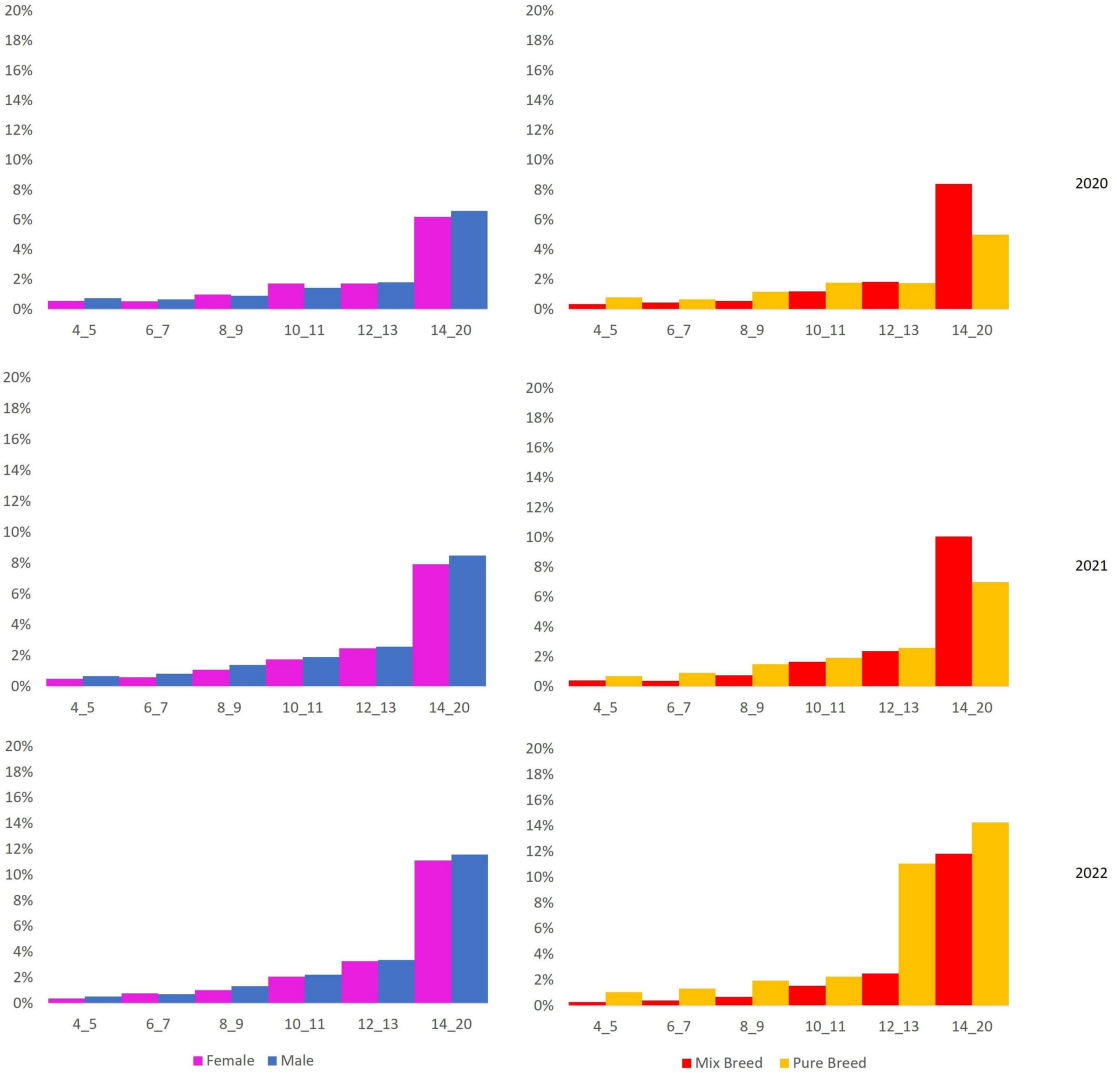
On the right: mortality rate stratified by sex (female/male) and age group (4–5 years, 6–7 years, 8–9 years, 10–11 years, 12–13 years, 14–20 years). On the left: mortality rate stratified by breed (purebred, not purebred) and age group (4–5 years, 6–7 years, 8–9 years, 10–11 years, 12–13 years, 14–20 years).

Then, mortality rates were calculated for 2020, 2021, and 2022, stratified by sex, breed, and age group (Figure 4).

The mortality rate increases with age in male and female dogs and purebred and not-purebred dogs, as naturally expected.

However, the mortality rate in male and not-purebred dogs is higher than in female and purebred dogs, respectively. Raw regional mortality (deaths/1000) is 30.05 (95%CI: from 29.6 to 30.5). Stratified mortality rates are reported in Table 1. All standardized mortality rates (SMRs) for all Ligurian municipalities are reported in Table S1, Supplementary material.

**Table 1:** Mortality rate (deaths/1000) stratified by breed and sex.

Breed	Sex	Deaths (n)	Population (n)	Mortality Rate IC 95%
Not Purebred	Female	3,350	107,406	31.19 (95%CI: from 30.13 to 32.24)
	Male	3,992	104,832	38.08 (95%CI: from 36.89 to 39.26)
Purebred	Female	4,615	176,447	26.15 (95%CI: from 25.4 to 26.9)
	Male	5,550	193,879	28.62 (95%CI: from 27.87 to 29.37)

**Figure 5:**
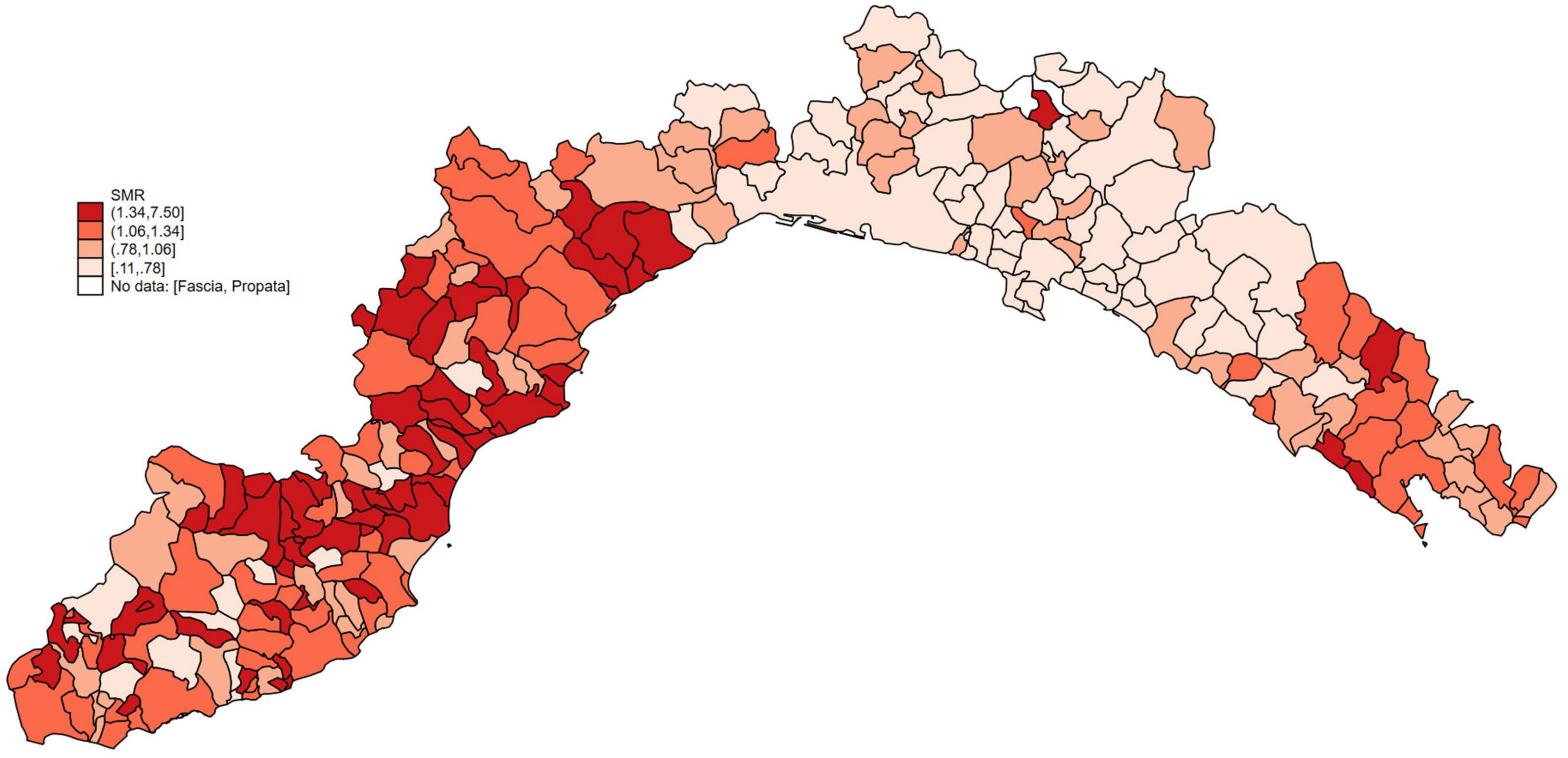
Heat map of Ligurian cities representing SMRs. Data were classified by quartiles (I, II, III, IV). Round brackets mean that value is not included in the range, while square brackets mean that value is included in the range.

Then, SMRs, stratified by sex and breed, were calculated and are presented in the heat map in Figure 5. As the map reveals, the highest SMR values are present in the western area of the region.

### 3.3 Risk factors: univariate and multivariate analysis

**Figure 6:**
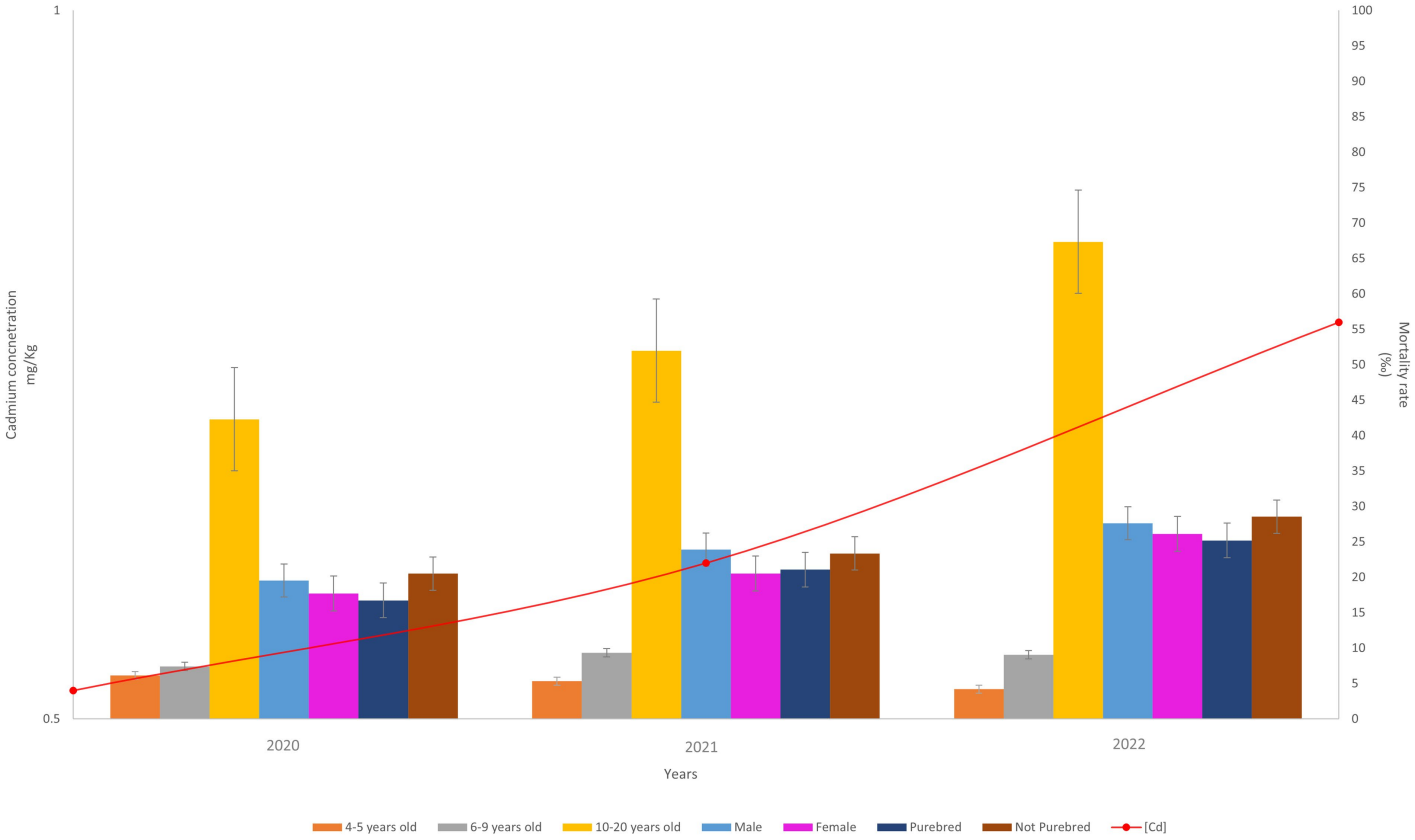
On the right y-axis: trend of the average of the mortality rate (deaths/1000). On the left y-axis: trend of the cadmium concentration (mean) during 2020–2022.

The graph (Figure 6) shows that the means of cadmium pollution extracted from WB and the dog mortality rates, expressed as per mil, have the same trends, they increase over time. In particular, cadmium concentration increased from 0.52 mg/kg in 2020 to 0.78 mg/kg in 2022. The overall mortality rate increased from 24.6‰ (95%CI: from 25.2 to 23.9) in 2020 to 36.1‰ (95%CI: from 35.5 to 37.0) in 2022. The female and male mortality rates also increased, from 17.7‰ (95%CI: from 14.1 to 21.3) in 2020 to 26.1‰ (95%CI: from 21.6 to 30.6) in 2022 and from 19.5‰ (95%CI: from 15.2 to 23.8) in 2020 to 27.6‰ (95%CI: from 23.6 to 31.7) in 2022, respectively. The trends in dog breeds are observed to be consistent with those in gender, with not-purebred breeds following the trend observed in male dogs and purebred breeds following the trend observed in female dogs. In the 10–20 years age group, mortality increased from 42.3‰ (95%CI: from 37.2 to 47.4) in 2020 to 67.3‰ (95%CI: from 60.5 to 74.2) in 2022.

Municipalities with higher mortality rates present significantly higher levels of cadmium, whereas no correlation between lead concentration and mortality was found. Passing from a low polluted area to a high polluted area, the mortality risk increases by 37% (95%CI: from 33 to 42%), while lead pollution does not present a risk factor. Male dogs have an 11% (95%CI: from 8 to 15%) higher risk of death than female dogs considering a multivariate model. Not-purebred dogs’ mortality risk is 26% (95%CI: from 22 to 30%) higher than purebred dogs in all models. In addition, mortality risk increases with age, at 64% (95%CI: from 46 to 83%) for the 6-9-years groups and 795% (95%CI: from 708 to 891%) for the oldest age group.

The two main causes of death for all Ligurian dogs are cancer and euthanasia. Among male dogs, skin tissues, the penis and testicles are the most affected by cancer, while, for female dogs, skin tissues and mammary glands are most affected, as reported in the Supplementary material (Figure S1 in Supplementary material). By cross-referencing the types of cancer with cadmium concentration, statistical elaboration shows that in most polluted areas, dogs are 68% (95%CI: from 60 to 74%) more likely to develop cancers in skin tissues, 96.4% (95%CI: from 94.5 to 97.6%) in the penis and testicles, and 86% (95%CI: from 82 to 89%) in mammary glands.

## 4 Discussion

Animal studies are a useful tool to ordinary life and to better and quickly understand human disease. In fact, dog mortality tables are used to estimate the typical remaining lifespan of adult dogs in canine adoption centers and to make decisions, in case of an adoption, on whether to link certain owners with certain dog breeds (1). In addition, in a One Health approach, animal studies provide another point of view in the study of human and environmental issues. Epidemiological studies conducted in humans and in the veterinary domain have shown that dogs are good sentinel animals for the study of the association of onset of diseases and/or mortality following chronic exposure to heavy metals (Cd, Pb), especially in comparative oncology (28, 56, 57).

In our study, the data show that there is no difference in the patterns in all 3 years of the study period in the region investigated. Populations remain constant and equally distributed over the age groups. Death counts (Figures 2, 3) and mortality rates (Figure 4) increase with age, as naturally expected. These trends are common in female and male dogs and not-purebred and purebred dogs (Table 1).

Female dogs were found to have a lower mortality rate compared to male dogs; this result is in agreement with that reported by Teng et al. (1). However, the mortality difference between genders may be influenced by the neutering status of dogs. Neutering has a dual impact on the life expectancy of dogs. On the one hand, it is a protective measure against reproductive organ tumors, but on the other hand, it could also increase the risk of certain cancers, such as lymphoma and hemangiosarcoma, especially in females. On the recommendation of veterinary professionals, neutering of female dogs often occurs before or soon after the first estrus cycle, which is why it may occur earlier in females compared to males. Therefore, in order to gain a better understanding of the complex issue of the gender gap in lifespan, the neutering status and associated data play a key role and need to be collected in further dog registers.

Comparing Ligurian mortality to the mortality rates obtained from the Umbria Registry, our mortality rates are smaller, especially in not-purebred dogs (9). As the authors suggest (9), mastiff dogs, which are typically purebred, are often employed for hunting purposes. As such, larger dogs may be more susceptible to environmental pollution compared to smaller “toy dogs” (which are often purebred) that are typically kept indoors or in less-exposed environments. A lifespan difference between small and molosser breeds has been demonstrated by two clinical studies (5, 58), but no significant difference was reported between different breeds within the same size category. So, assuming that the majority of not purebred dogs are of large size, as proposed by other authors (9), our findings are consistent with previous literature, suggesting that smaller dogs have a longer life expectancy. Nevertheless, a more comprehensive dog registry database that includes information on breed and size could facilitate further investigations into the differences in lifespan across various size categories of dogs.

Since in most of the Ligurian municipalities, the number of deaths was small and the variance was relatively small, we estimated SMRs using the indirect method, considering the regional mortality as a reference (59). All SMRs are reported in Table S1, in the Supplementary material.

To understand the relationship between environmental pollution and dog mortality, we have compared the mortality of dogs living in areas with different levels of heavy metal pollution. Considering that health issues and diseases arising from air pollution, especially the accumulation of metals, appear only after long time and require biological processes, we opted to collect data on environmental pollution over 10 years and before the examined period for mortality assessments (44). Our data analysis revealed that dogs living in more cadmium-polluted areas had higher mortality rates than those living in less-polluted areas. The values of SMRs shown in Figure 5 are higher in the provinces of Savona and La Spezia, where higher concentrations of cadmium in WB were also found (Figure 1A). Municipalities with higher lead pollution are widespread throughout the region (Figure 1B). The higher pollution levels in Savona and La Spezia could be due to the presence of active thermoelectric power plants, military activity, intensive port activities, and LNG regasification terminals.

In univariate Poisson, there is a significant correlation between cadmium concentration and dog mortality, IRR = 1.37 (95%CI: from 1.33 to 1.42). As observed by Franzoni et al. (28), this suggests that the animals are being chronically exposed to a heavy metal that can accumulate in target organs such as the liver and kidneys over time, potentially leading to the development of lethal pathologies and, in turn, higher mortality values. Nonetheless, since cadmium pollution is not the sole contributing factor to dog mortality, we performed a multivariate Poisson regression to predict dogs’ mortality, and this association remains significant in multivariate Poisson regression adjusted for sex, breed, and age.

According to Jianming Xu et al. (24), the primary sources of cadmium include non-ferrous metal smelting, coal consumption, biomass burning, non-metallic mineral manufacturing, and liquid fuel combustion. In 2019 and 2020, when pandemic restrictions were in place, industrial activities were reduced, resulting in lower cadmium emissions. Therefore, assuming that the cadmium bioaccumulated in 2020 represents the baseline level, the increase in the mean cadmium absorbed by WB (Figure 1A) is consistent with the actual scenario. However, the principal finding from the graph in Figure 4 is that the average mortality of dogs in the 10–20 years age group shows an upward trend, along with the increase in cadmium concentration, from 2020 to 2022. This could suggest that dogs exposed to cadmium contamination for at least 10 years may have receptors that are highly sensitive to cadmium stress, resulting in a slight deterioration of their health and ultimately leading to fatal events.

Some authors (60) think that even low concentrations of heavy metal ions taken in youth might become toxic with age and trigger degenerative diseases associated with protein misfolding. Once metal complex protein–cadmium is formed, the complex could affect both the native refolding pathway of chaperone-repaired (unfolded) aggregated proteins and also directly inactivate the chaperone machinery. Thus, elevated cellular metal concentrations would soon lead to cell death, overwhelming and neutralizing the protein quality-control machinery (60–62). So, even low levels of heavy metals may be sufficient to induce cell death in aged cells, which have low levels of chaperones.

## 5 Conclusion

Our study has demonstrated, for the first time, that cadmium pollution is significantly correlated with a higher risk of mortality.

WB, as consolidated in scientific literature, are good environmental *biomonitors* and are therefore useful to monitor persistent pollutants in the habitats of ungulates (10–13, 17–20). Biomonitor data shows that 39% of the Ligurian municipalities investigated have cadmium concentrations above the EU limit (0.50 mg/kg), while all investigated municipalities have lead concentrations above the EU limit (0.10 mg/kg). Our findings indicate that WB are chronically exposed to cadmium (Figure 6), resulting in the accumulation of heavy metals in target organs over several years, as evidenced by a comparison of data from 2020 and 2022.

We have also identified a difference in mortality risk between male and female dogs, with males at higher risk. Additionally, we have observed a 26% higher mortality risk for non-purebred dogs compared to purebred dogs in all models, and this could be affected by dog size, as reported by others (5, 9, 58). The data highlighted that metal pollution affects dogs’ health not in early life but after at least 10 years of heavy metal accumulation. In cadmium-polluted municipalities, dogs are more likely to develop skin, penis, testicular, and mammary gland cancers compared with low-pollution areas. However, the data do not show a direct correlation between mortality and pollution. Further studies, including a more complex pollution panel, will facilitate a better understanding of the link between environmental pollution and pet mortality.

Using a Poisson regression model, we identified specific factors that contribute to dog mortality and provided insights into potential solutions for mitigating the impact of environmental pollution on animal and human health. The results show that risk factors include cadmium pollution, gender, breed, and age. Lead pollution, which is widespread throughout the region with almost equal distribution, could not be considered as a significant parameter and it was therefore ignored in the multivariable models.

Close contact between rural and urban areas, which is very typical in Liguria, is a unique geographical conformation that leads to a miscellany of pets and sylvatic animals. In conclusion, this study highlights the importance of conducting health research on pets and using them as sentinels for human health. Our preliminary findings have substantial implications not only for the health and well-being of dogs but also for human health, providing a basis for future studies involving both animal and human health.

## Data availability statement

The datasets generated during and/or analyzed during the current study are available from the corresponding author upon reasonable request.

## Ethics statement

The animal studies were approved by Comitato Etico dell’Istituto Zooprofilattico Sperimentale del Piemonte Liguria e Valle d’Aosta (IZS PLVA). The studies were conducted in accordance with the local legislation and institutional requirements. Written informed consent was obtained from the owners for the participation of their animals in this study.

## Author contributions

RG: Conceptualization, Data curation, Formal analysis, Investigation, Methodology, Software, Supervision, Validation, Visualization, Writing – original draft, Writing – review & editing. MC: Conceptualization, Software, Supervision, Validation, Writing – review & editing. VCo: Funding acquisition, Writing – review & editing. VCi: Formal analysis, Supervision, Writing – review & editing. BV: Validation, Writing – review & editing. ER: Funding acquisition, Investigation, Project administration, Resources, Supervision, Validation, Writing – review & editing.

## Footnotes

^1^ https://www.ons.gov.uk
