# Supplementary material for: Mortality and heavy metals environmental exposure: a study in dogs
Source: Front Vet Sci. 2024 Jan 5;10:1297311. doi: 10.3389/fvets.2023.1297311 (PMC10796622; doi:10.3389/fvets.2023.1297311)

**Supplementary Material**

**Table S1**: Mortality rates for all Ligurian municipalities.

| **Municipality** | **SMRs** | **(95% CI)** | |
| --- | --- | --- | --- |
| AIROLE | 2.66 | 1.22 | 5.05 |
| ALASSIO | 0.94 | 0.76 | 1.15 |
| ALBENGA | 1.53 | 1.39 | 1.68 |
| ALBISOLA SUPERIORE | 1.46 | 1.25 | 1.71 |
| ALBISSOLA MARINA | 1.64 | 1.32 | 2.01 |
| ALTARE | 1.64 | 1.22 | 2.16 |
| AMEGLIA | 1.18 | 0.95 | 1.44 |
| ANDORA | 1.22 | 1.02 | 1.44 |
| APRICALE | 2.18 | 1.35 | 3.33 |
| AQUILA D'ARROSCIA | 2.89 | 1.50 | 5.05 |
| ARCOLA | 1.18 | 1.03 | 1.35 |
| ARENZANO | 0.77 | 0.60 | 0.98 |
| ARMO | 2.64 | 0.97 | 5.75 |
| ARNASCO | 2.19 | 1.43 | 3.20 |
| AURIGO | 0.81 | 0.35 | 1.60 |
| AVEGNO | 0.53 | 0.33 | 0.80 |
| BADALUCCO | 1.86 | 1.34 | 2.50 |
| BAJARDO | 1.77 | 0.99 | 2.93 |
| BALESTRINO | 1.14 | 0.72 | 1.70 |
| BARDINETO | 1.83 | 1.32 | 2.46 |
| BARGAGLI | 0.60 | 0.45 | 0.79 |
| BERGEGGI | 1.31 | 0.77 | 2.06 |
| BEVERINO | 1.14 | 0.85 | 1.49 |
| BOGLIASCO | 0.96 | 0.68 | 1.32 |
| BOISSANO | 0.87 | 0.64 | 1.17 |
| BOLANO | 0.92 | 0.77 | 1.09 |
| BONASSOLA | 1.33 | 0.78 | 2.14 |
| BORDIGHERA | 1.46 | 1.26 | 1.69 |
| BORGHETTO D'ARROSCIA | 1.28 | 0.66 | 2.23 |
| BORGHETTO DI VARA | 0.88 | 0.44 | 1.57 |
| BORGHETTO SANTO SPIRITO | 1.12 | 0.82 | 1.48 |
| BORGIO VEREZZI | 1.82 | 1.33 | 2.45 |
| BORGOMARO | 1.38 | 0.92 | 1.99 |
| BORMIDA | 1.06 | 0.53 | 1.89 |
| BORZONASCA | 0.50 | 0.32 | 0.75 |
| BRUGNATO | 1.04 | 0.59 | 1.69 |
| BUSALLA | 0.59 | 0.40 | 0.83 |
| CAIRO MONTENOTTE | 1.34 | 1.19 | 1.50 |
| CALICE AL CORNOVIGLIO | 1.19 | 0.87 | 1.60 |
| CALICE LIGURE | 1.77 | 1.32 | 2.31 |
| CALIZZANO | 1.23 | 0.87 | 1.70 |
| CAMOGLI | 0.46 | 0.30 | 0.67 |
| CAMPO LIGURE | 0.70 | 0.45 | 1.04 |
| CAMPOMORONE | 0.66 | 0.47 | 0.89 |
| CAMPOROSSO | 1.23 | 1.02 | 1.47 |
| CARASCO | 0.70 | 0.47 | 1.02 |
| CARAVONICA | 1.85 | 0.95 | 3.23 |
| CARCARE | 1.51 | 1.24 | 1.82 |
| CARRO | 0.37 | 0.12 | 0.87 |
| CARRODANO | 1.11 | 0.51 | 2.10 |
| CASANOVA LERRONE | 1.36 | 0.88 | 2.00 |
| CASARZA LIGURE | 0.65 | 0.50 | 0.84 |
| CASELLA | 0.69 | 0.47 | 0.97 |
| CASTEL VITTORIO | 2.36 | 1.44 | 3.64 |
| CASTELBIANCO | 1.12 | 0.51 | 2.12 |
| CASTELLARO | 0.83 | 0.50 | 1.28 |
| CASTELNUOVO MAGRA | 1.23 | 1.05 | 1.44 |
| CASTELVECCHIO DI ROCCA BARBENA | 0.90 | 0.24 | 2.30 |
| CASTIGLIONE CHIAVARESE | 0.74 | 0.46 | 1.11 |
| CELLE LIGURE | 1.74 | 1.44 | 2.09 |
| CENGIO | 1.14 | 0.85 | 1.49 |
| CERANESI | 0.60 | 0.42 | 0.83 |
| CERIALE | 1.62 | 1.31 | 1.97 |
| CERIANA | 0.68 | 0.43 | 1.03 |
| CERVO | 1.31 | 0.78 | 2.08 |
| CESIO | 2.32 | 1.41 | 3.58 |
| CHIAVARI | 0.73 | 0.62 | 0.86 |
| CHIUSANICO | 1.08 | 0.63 | 1.73 |
| CHIUSAVECCHIA | 1.48 | 0.71 | 2.73 |
| CICAGNA | 0.83 | 0.51 | 1.29 |
| CIPRESSA | 1.20 | 0.78 | 1.77 |
| CISANO SUL NEVA | 2.08 | 1.67 | 2.55 |
| CIVEZZA | 2.00 | 1.29 | 2.95 |
| COGOLETO | 0.51 | 0.38 | 0.67 |
| COGORNO | 0.71 | 0.52 | 0.95 |
| COREGLIA LIGURE | 0.95 | 0.20 | 2.77 |
| COSIO D'ARROSCIA | 1.68 | 0.81 | 3.09 |
| COSSERIA | 1.21 | 0.87 | 1.63 |
| COSTARAINERA | 1.66 | 1.03 | 2.54 |
| CROCEFIESCHI | 1.11 | 0.57 | 1.93 |
| DAVAGNA | 0.62 | 0.40 | 0.90 |
| DEGO | 1.12 | 0.89 | 1.39 |
| DEIVA MARINA | 1.49 | 0.88 | 2.35 |
| DIANO ARENTINO | 1.16 | 0.68 | 1.86 |
| DIANO CASTELLO | 0.96 | 0.68 | 1.32 |
| DIANO MARINA | 1.50 | 1.21 | 1.83 |
| DIANO SAN PIETRO | 1.24 | 0.84 | 1.75 |
| DOLCEACQUA | 1.22 | 0.92 | 1.59 |
| DOLCEDO | 1.48 | 1.08 | 1.99 |
| ERLI | 1.93 | 0.88 | 3.67 |
| FASCIA | 0.00 | 0.00 | 2.75 |
| FAVALE DI MALVARO | 0.46 | 0.09 | 1.34 |
| FINALE LIGURE | 1.46 | 1.27 | 1.68 |
| FOLLO | 1.27 | 1.06 | 1.51 |
| FONTANIGORDA | 0.98 | 0.27 | 2.50 |
| FRAMURA | 0.50 | 0.18 | 1.08 |
| GARLENDA | 1.38 | 0.95 | 1.93 |
| GENOVA | 0.64 | 0.62 | 0.66 |
| GIUSTENICE | 1.20 | 0.80 | 1.74 |
| GIUSVALLA | 1.08 | 0.59 | 1.81 |
| GORRETO | 1.04 | 0.03 | 5.82 |
| IMPERIA | 1.31 | 1.21 | 1.41 |
| ISOLA DEL CANTONE | 0.57 | 0.31 | 0.96 |
| ISOLABONA | 1.72 | 1.12 | 2.52 |
| LA SPEZIA | 1.32 | 1.24 | 1.40 |
| LAIGUEGLIA | 1.07 | 0.61 | 1.74 |
| LAVAGNA | 0.77 | 0.61 | 0.95 |
| LEIVI | 0.63 | 0.46 | 0.85 |
| LERICI | 1.09 | 0.92 | 1.28 |
| LEVANTO | 1.26 | 0.99 | 1.58 |
| LOANO | 1.39 | 1.19 | 1.61 |
| LORSICA | 1.02 | 0.49 | 1.88 |
| LUCINASCO | 1.69 | 0.77 | 3.20 |
| LUMARZO | 0.69 | 0.42 | 1.05 |
| LUNI | 1.14 | 0.97 | 1.33 |
| MAGLIOLO | 1.27 | 0.89 | 1.75 |
| MAISSANA | 0.92 | 0.44 | 1.69 |
| MALLARE | 1.13 | 0.76 | 1.61 |
| MASONE | 1.17 | 0.86 | 1.55 |
| MASSIMINO | 4.27 | 2.33 | 7.16 |
| MELE | 0.53 | 0.35 | 0.77 |
| MENDATICA | 1.11 | 0.36 | 2.60 |
| MEZZANEGO | 0.71 | 0.39 | 1.19 |
| MIGNANEGO | 0.73 | 0.53 | 0.99 |
| MILLESIMO | 1.16 | 0.87 | 1.51 |
| MIOGLIA | 1.61 | 1.08 | 2.31 |
| MOCONESI | 0.82 | 0.53 | 1.20 |
| MOLINI DI TRIORA | 1.43 | 0.93 | 2.09 |
| MONEGLIA | 0.76 | 0.50 | 1.11 |
| MONTALTO CARPASIO | 0.13 | 0.00 | 0.70 |
| MONTEBRUNO | 0.38 | 0.05 | 1.36 |
| MONTEGROSSO PIAN LATTE | 7.97 | 3.98 | 14.26 |
| MONTEROSSO AL MARE | 0.99 | 0.54 | 1.67 |
| MONTOGGIO | 0.63 | 0.42 | 0.90 |
| MURIALDO | 1.64 | 1.01 | 2.50 |
| NASINO | 0.95 | 0.20 | 2.79 |
| NE | 0.68 | 0.44 | 0.99 |
| NEIRONE | 1.05 | 0.64 | 1.63 |
| NOLI | 1.54 | 1.17 | 1.99 |
| OLIVETTA SAN MICHELE | 1.68 | 0.62 | 3.66 |
| ONZO | 0.91 | 0.25 | 2.32 |
| ORCO FEGLINO | 0.90 | 0.49 | 1.52 |
| ORERO | 0.19 | 0.00 | 1.07 |
| ORTOVERO | 1.33 | 0.93 | 1.83 |
| OSIGLIA | 1.66 | 0.80 | 3.06 |
| OSPEDALETTI | 1.29 | 0.99 | 1.65 |
| PALLARE | 2.16 | 1.60 | 2.85 |
| PERINALDO | 0.75 | 0.46 | 1.15 |
| PIANA CRIXIA | 1.13 | 0.77 | 1.60 |
| PIETRA LIGURE | 1.29 | 1.04 | 1.59 |
| PIETRABRUNA | 1.41 | 0.70 | 2.51 |
| PIEVE DI TECO | 1.54 | 1.11 | 2.07 |
| PIEVE LIGURE | 0.63 | 0.37 | 0.99 |
| PIGNA | 0.97 | 0.48 | 1.74 |
| PIGNONE | 0.80 | 0.32 | 1.64 |
| PLODIO | 0.97 | 0.61 | 1.47 |
| POMPEIANA | 1.16 | 0.65 | 1.92 |
| PONTEDASSIO | 1.46 | 1.14 | 1.84 |
| PONTINVREA | 2.02 | 1.48 | 2.71 |
| PORNASSIO | 1.68 | 1.01 | 2.62 |
| PORTOFINO | 0.66 | 0.08 | 2.37 |
| PORTOVENERE | 1.29 | 0.93 | 1.74 |
| PRELÀ | 1.21 | 0.64 | 2.07 |
| PROPATA | 0.00 | 0.00 | 0.99 |
| QUILIANO | 1.13 | 0.95 | 1.34 |
| RANZO | 1.60 | 0.95 | 2.53 |
| RAPALLO | 0.68 | 0.59 | 0.79 |
| RECCO | 0.60 | 0.45 | 0.79 |
| REZZO | 1.13 | 0.56 | 2.02 |
| REZZOAGLIO | 0.85 | 0.50 | 1.36 |
| RIALTO | 0.61 | 0.24 | 1.25 |
| RICCÒ DEL GOLFO DI SPEZIA | 1.20 | 0.95 | 1.50 |
| RIOMAGGIORE | 1.86 | 1.17 | 2.82 |
| RIVA LIGURE | 0.79 | 0.52 | 1.15 |
| ROCCAVIGNALE | 1.48 | 0.89 | 2.30 |
| ROCCHETTA DI VARA | 1.61 | 1.06 | 2.34 |
| ROCCHETTA NERVINA | 2.55 | 1.36 | 4.37 |
| RONCO SCRIVIA | 0.71 | 0.49 | 0.99 |
| RONDANINA | 1.24 | 0.26 | 3.62 |
| ROSSIGLIONE | 0.54 | 0.30 | 0.91 |
| ROVEGNO | 0.69 | 0.28 | 1.43 |
| SAN BARTOLOMEO AL MARE | 1.38 | 1.01 | 1.85 |
| SAN BIAGIO DELLA CIMA | 1.21 | 0.80 | 1.76 |
| SAN COLOMBANO CERTENOLI | 0.53 | 0.36 | 0.75 |
| SAN LORENZO AL MARE | 1.22 | 0.72 | 1.92 |
| SANREMO | 1.22 | 1.14 | 1.30 |
| SANT'OLCESE | 0.84 | 0.67 | 1.05 |
| SANTA MARGHERITA LIGURE | 0.65 | 0.48 | 0.86 |
| SANTO STEFANO AL MARE | 1.25 | 0.80 | 1.86 |
| SANTO STEFANO D'AVETO | 1.06 | 0.69 | 1.56 |
| SANTO STEFANO DI MAGRA | 1.06 | 0.93 | 1.22 |
| SARZANA | 1.15 | 1.04 | 1.27 |
| SASSELLO | 0.94 | 0.68 | 1.26 |
| SAVIGNONE | 0.59 | 0.38 | 0.87 |
| SAVONA | 1.37 | 1.27 | 1.47 |
| SEBORGA | 1.91 | 1.02 | 3.26 |
| SERRA RICCÒ | 0.77 | 0.62 | 0.94 |
| SESTA GODANO | 1.40 | 1.00 | 1.92 |
| SESTRI LEVANTE | 0.93 | 0.79 | 1.10 |
| SOLDANO | 0.95 | 0.56 | 1.50 |
| SORI | 0.75 | 0.51 | 1.05 |
| SPOTORNO | 1.44 | 1.09 | 1.86 |
| STELLA | 1.69 | 1.41 | 2.02 |
| STELLANELLO | 1.17 | 0.77 | 1.69 |
| TAGGIA | 1.09 | 0.92 | 1.27 |
| TERZORIO | 1.40 | 0.29 | 4.09 |
| TESTICO | 0.47 | 0.06 | 1.70 |
| TIGLIETO | 1.04 | 0.50 | 1.92 |
| TOIRANO | 1.36 | 1.04 | 1.75 |
| TORRIGLIA | 0.93 | 0.70 | 1.22 |
| TOVO SAN GIACOMO | 1.16 | 0.91 | 1.47 |
| TRIBOGNA | 1.14 | 0.52 | 2.16 |
| TRIORA | 0.82 | 0.30 | 1.78 |
| URBE | 1.02 | 0.59 | 1.63 |
| USCIO | 0.55 | 0.32 | 0.90 |
| VADO LIGURE | 1.28 | 1.06 | 1.52 |
| VALBREVENNA | 0.63 | 0.33 | 1.10 |
| VALLEBONA | 1.33 | 0.90 | 1.90 |
| VALLECROSIA | 1.06 | 0.84 | 1.32 |
| VARAZZE | 1.44 | 1.26 | 1.63 |
| VARESE LIGURE | 0.64 | 0.45 | 0.90 |
| VASIA | 2.10 | 1.15 | 3.53 |
| VENDONE | 2.82 | 1.92 | 4.00 |
| VENTIMIGLIA | 1.13 | 1.01 | 1.25 |
| VERNAZZA | 1.82 | 1.06 | 2.91 |
| VESSALICO | 1.21 | 0.52 | 2.39 |
| VEZZANO LIGURE | 1.17 | 0.98 | 1.39 |
| VEZZI PORTIO | 0.86 | 0.49 | 1.40 |
| VILLA FARALDI | 1.78 | 0.95 | 3.05 |
| VILLANOVA D'ALBENGA | 1.33 | 1.03 | 1.68 |
| VOBBIA | 0.71 | 0.26 | 1.55 |
| ZIGNAGO | 1.26 | 0.78 | 1.92 |
| ZOAGLI | 0.78 | 0.52 | 1.14 |
| ZUCCARELLO | 0.62 | 0.17 | 1.59 |

**Table S2**: List of municipalities for which information on the concentration of Cd and/or Pb extracted from the target organs of WB is missing.

| **Municipality** | **Province** |
| --- | --- |
| AIROLE | Imperia |
| ALBISSOLA MARINA | Savona |
| ARMO | Imperia |
| AVEGNO | Genova |
| BADALUCCO | Imperia |
| BOGLIASCO | Genova |
| BOLANO | La Spezia |
| BONASSOLA | La Spezia |
| BORDIGHERA | Imperia |
| BORGHETTO SANTO SPIRITO | Savona |
| CAMOGLI | Genova |
| CASTELLARO | Imperia |
| CASTELNUOVO MAGRA | La Spezia |
| CERIANA | Imperia |
| CESIO | Imperia |
| CHIUSAVECCHIA | Imperia |
| CIVEZZA | Imperia |
| COSIO D'ARROSCIA | Imperia |
| COSSERIA | Savona |
| COSTARAINERA | Imperia |
| CROCEFIESCHI | Genova |
| DIANO ARENTINO | Imperia |
| DIANO MARINA | Imperia |
| DIANO SAN PIETRO | Imperia |
| DOLCEACQUA | Imperia |
| FASCIA | Genova |
| FOLLO | La Spezia |
| LAVAGNA | Genova |
| LUCINASCO | Imperia |
| LUNI | La Spezia |
| MONTEROSSO AL MARE | La Spezia |
| PIETRABRUNA | Imperia |
| PIEVE LIGURE | Genova |
| POMPEIANA | Imperia |
| PORTOFINO | Genova |
| PROPATA | Genova |
| RIOMAGGIORE | La Spezia |
| RIVA LIGURE | Imperia |
| ROCCHETTA NERVINA | Imperia |
| RONDANINA | Genova |
| SAN BIAGIO DELLA CIMA | Imperia |
| SAN LORENZO AL MARE | Imperia |
| SANTAMARGHERITA LIGURE | Genova |
| SANTO STEFANO AL MARE | Imperia |
| SEBORGA | Imperia |
| TAGGIA | Imperia |
| TERZORIO | Imperia |
| TRIBOGNA | Genova |
| VALLECROSIA | Imperia |
| VASIA | Imperia |
| VERNAZZA | La Spezia |

**Figure S1**: Tumour sites of female (A) and male (B) dogs collected during 2020-2022.

A)
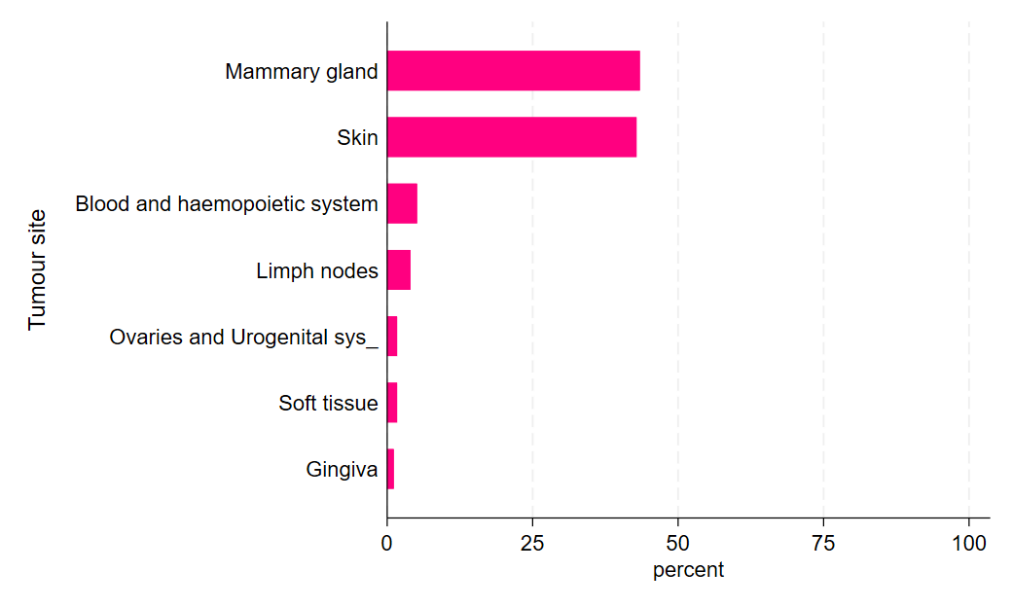


B)
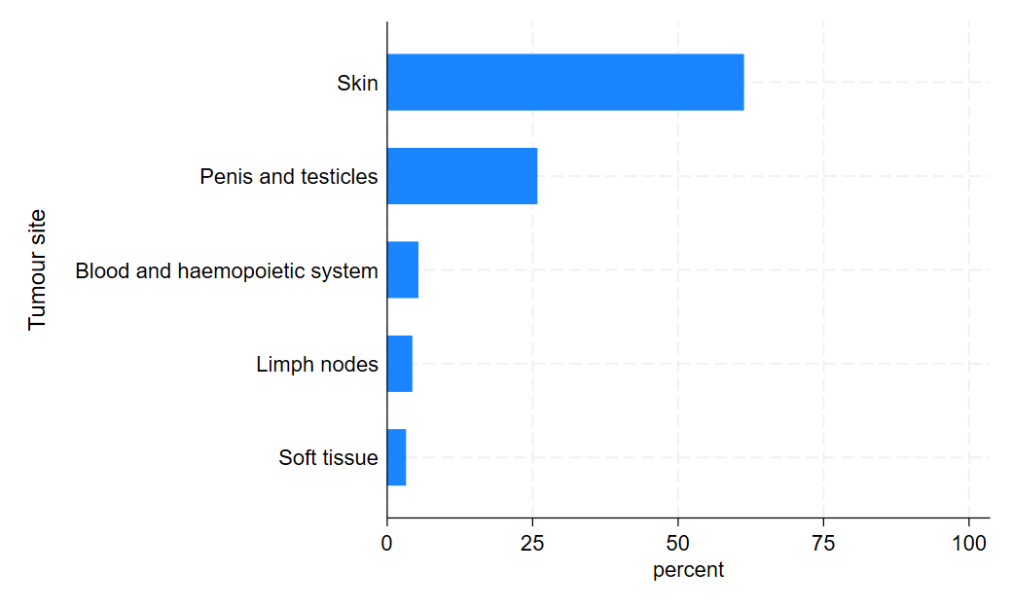

Supplement: Supplementary file 1 [file Data_Sheet_1.docx]
